# High efficacy therapy to prevent the formation of meningeal tertiary lymphoid organs after CXCL13 index screening in early multiple sclerosis

**DOI:** 10.3389/fnins.2025.1558810

**Published:** 2025-03-17

**Authors:** Ana C. Londoño, Carlos A. Mora

**Affiliations:** ^1^Instituto Neurologico de Colombia (INDEC), Medellin, Colombia; ^2^Retired (2022), Medellin, Colombia; ^3^Department of Medicine, Neurology Unit, Virginia Tech Carilion School of Medicine, Roanoke, VA, United States

**Keywords:** CXCL13, ICXCL13, high efficacy therapy, multiple sclerosis, neuroinflammation, tertiary lymphoid organ

## Abstract

Postmortem studies have shown the presence of subpial inflammation with tertiary lymphoid organs (TLO) in the meninges of patients with progressive multiple sclerosis, playing an important role in the pathophysiology of the disease. The chemokine (C-X-C motif) ligand 13 (CXCL13) induces the formation of these lymphoid organs, thus promoting activity of disease. The progression to disability in multiple sclerosis has been reduced, thanks to the effect of disease modifying therapy. However, despite advances in the treatment of disease with immunomodulatory agents, we still lack specific laboratory biomarkers that could indicate the state of activity of disease, either at time of diagnosis or when escalation therapy seems to be mandatory. In patients with multiple sclerosis, MRI studies have not demonstrated the presence of TLO in the CNS, so far. The determination of the CXCL13 index (ICXCL 13), in clinical specimens, could become a reliable biomarker for the verification of the presence and activity of the TLO, thus contributing to improving therapy outcome, with high efficacy therapy, in the clinical setting.

## 1 Introduction

Multiple sclerosis (MS) is a neurological disease that results from dysfunction of the immune system, affecting individuals between the age of 18 and 65. The disease is mediated by B and T lymphocytes. Although it responds to treatment with disease modified therapy (DMT) drugs, it could lead to progressive disability during lifetime span if the therapeutic approach is not optimal. It has been a matter of controversy whether patients with MS, right after diagnosis, should be started on ‘escalation’ therapy with DMT or begin at once treatment with high efficacy therapy (HET). Although early intervention with high-efficacy disease modifying/induction therapy may offer the best risk/benefit profile for patients with relapsing–remitting MS (RRMS) who are young and have active disease (with reduction of risk of progression to disability and conversion to progressive forms of the disease), the use of HET drugs that achieve sustained immunosuppression, or induction drugs that achieve short term immunosuppression, may complicate with adverse effects such as opportunistic infection, cancer or autoimmune disease (Hartung et al., 2021; Coveñas et al., 2024; Edan and Le Page, 2023).

In MS, the meninges experiment the distribution of the inflammatory process through the formation of cellular aggregates known as tertiary lymphoid organs (TLO), which mimic the structure and function of the secondary lymphoid structures (SLO) described in other organs (Shipman et al., 2017; Kee et al., 2022). However, it is not clear how early these structures develop, and when they will participate actively, in the disease pathogenesis (Pitzalis et al., 2014). TLO are structures that regulate and promote the development of immune cells to mediate infectious processes or lead to the development of autoimmune, inflammatory or neoplastic disease in several organs (Shipman et al., 2017). TLO can remain active for several weeks due to the lack of recirculation of immune cells from the periphery (Pitzalis et al., 2014).

The chemokine(C-X-C) ligand 13 (CXCL13) regulates traffic of lymphocytes and their distribution in the germinal centers in lymphoid organs, including the TLO, on top of being a potent chemoattractant to the central nervous system (Londoño and Mora, 2018). In this perspective article, we focus on highlighting the importance of the determination of the chemokine (C-X-C) Ligand 13 index (ICXCL13) as an indicator of the presence of the meningeal tertiary lymphoid organ (mTLO) in patients with MS, since the presence of CXCL13 induces the formation of the germinal centers (GC) in the corresponding TLO of patients with this condition. Since TLO contributes to the progression of disease in several autoimmune diseases, blocking the formation of mTLO could contribute to slowing down progression of disease.

## 2 The meningeal tertiary lymphoid organs are independent from the peripheral circulation immunity

### 2.1 Meninges, tertiary lymphoid organ, and neuroinflammation

Immune cells that populate the healthy meninges display high levels of diversity, being able to modify their behavior with aging and during neurodegenerative and neuro-inflammatory conditions (Alves de Lima et al., 2020). Meningeal B cells are phenotypically like the bone marrow B cells (Brioschi et al., 2021). Studies in animal models have demonstrated that these lymphoid cells come from the bone marrow niches of calvaria (BMNc) and not from the blood circulation (Brioschi et al., 2021). The BMNc communicates with the underlying duramater through ossified vascular channels (Herrisson et al., 2018; Cai et al., 2019; Yao et al., 2018). Spinal fluid could reach the BMNc, thus, contributing to the regulation of myelopoiesis with a subsequent exit of myeloid cells to the meninges during both physiologic and pathologic conditions (Mazzitelli et al., 2022). The meninges host a diverse network of immune cells between the cerebral parenchyma, the glymphatic system, and the different meningeal layers, creating an important interphase between the periphery and the CNS parenchyma (Korin et al., 2017; Filiano et al., 2017; Mrdjen et al., 2018; Derk et al., 2021). Opposite to the dural vascular structure, the pia mater vessels have tight junctions that selectively transport molecules and immune cells in and out of the spinal fluid and the brain parenchyma. T-cells could transit from the peripheral circulation to the meningeal lymphatic vessels and continue to drain in the deep cervical lymphatic nodules where the cells may be exposed to antigen presentation and lymphocyte activation (Aspelund et al., 2015; Louveau et al., 2015; Da Mesquita et al., 2018; Louveau et al., 2018).

### 2.2 Immune protective barriers of the central nervous system

The vertebrate central nervous system (CNS) is protected by a highly specialized cellular and molecular structure, the meninges. In addition, brain parenchyma is protected by the blood-meningeal barrier, the blood-CSF barrier and the blood–brain barrier (Alves de Lima et al., 2020). Interactions among bone marrow niches of the calvaria (BMNc) with the CSF and the meninges may help to perpetuate and promote disease activity in MS (Alves de Lima et al., 2020). The re-discovery of meningeal lymphatics, which drain CNS-derived macromolecules and immune cells, has challenged the traditional concept of CNS immune privilege (Alves de Lima et al., 2020). A complete understanding of this interaction could provide useful information aimed at designing therapeutic strategies.

The mTLO has been associated with a more severe course of disease with faster progression toward disability (Kee et al., 2022). Also, mTLO participates in the propagation of neuroinflammation since they have been frequently found overlying subpial cortical lesions with presence of inflammation between the subpial surface and the subcortical white matter (Magliozzi et al., 2007). CNS immune surveillance seems to constantly occur in the meningeal compartment, which might act as an immunologically active barrier (Alves de Lima et al., 2020). Dysfunction of meningeal immunity may directly affect function in the aging brain (Alves de Lima et al., 2020). The meninges constitute an important checkpoint for T cell infiltration of the CNS during neuroinflammation (Alves de Lima et al., 2020). This is relevant since in individuals going on to develop MS, antibody producing B lineage cells invade the CNS predominantly at the time of, and triggered by, acute primary Epstein–Barr virus (EBV) infection (Otto et al., 2016).

The combined action of Th-17 cells, *β*-lymphotoxin, and the presence of EBV mRNA in memory B-cells of the GC could contribute to the genesis of the TLO (Kee et al., 2022; Serafini et al., 2016). In post-mortem MS cases, a role for virus-driven immunopathology is evident with the finding of tissue-resident memory (Trm) T cells, a subset of CD8+ T cells, that establish direct contact with EBV infected cells in the infiltrated MS white matter and meninges, including the TLO (Serafini et al., 2023). Some studies have shown that meningeal B cell follicles can harbor latent EBV transcripts (EBV-encoded small nuclear mRNA) and EBV latent membrane protein LMP-2A, thus, contributing to the propagation and expansion of TLOs in MS (Serafini et al., 2007; Serafini et al., 2010).

In CNS tissue obtained from deceased patients with MS, as well as from mouse models of MS, mTLO was identified within the deep sulci in the meninges of the brain, cerebellum, brainstem and spinal cord (Kee et al., 2022). Post-mortem studies in patients with SPMS disclosed mTLO that included presence of CD3+ T cells, CD20+ B cells, CD 138+ plasma cells and CD68+ macrophages (Serafini et al., 2004). Higher degrees of meningeal inflammation, in post-mortem MS tissue, are related to increased gene and protein expression of TNF-*α* and interferon (IFN)-*γ*, with higher expression levels in cases that had TLO (Gardner et al., 2013).

The presence of somatically hypermutated and clonally expanded B cells in the CSF, alongside the presence of oligoclonal bands (OCB), support the existence of an antigen-driven immune component in the pathogenesis of disease (Kee et al., 2022). TLO displaying GC are thought to support the production of autoantibodies in other autoimmune diseases such as rheumatoid arthritis, Sjogren’s syndrome (SS), Hashimoto’s thyroiditis (HT) and Graves’ disease (GD) (Kee et al., 2022). In patients with HT and GD, TLO demonstrating cardinal features of GC with distinct dark and light zones, high endothelial venules, follicular dendritic cells (FDC) and mantle zones, were identified in thyroid glands (Armengol et al., 2001). In salivary glands from patients with SS, TLO were found to have significantly higher levels of anti-Ro and anti-La autoantibodies (Salomonsson et al., 2003).

A clinical phenomenon called ‘progression independent of relapse activity’ (PIRA), begins early during MS and it has been proposed that most of the tissue injury associated with PIRA is started by autonomous mTLO, which may be present before onset of disease and may not respond to current therapy with DMT (Ransohoff, 2023).

### 2.3 Current validated biomarkers to determine progression or activity of MS

In MS, blood-based serum neurofilament light chain (sNfL), a neuron-specific cytoskeletal protein, was found elevated in specimens of patients even 6 years (4–10-year range) before the onset of clinical manifestations of disease (Bjornevik et al., 2020). sNfL levels strongly correlate with body mass index and blood volume, on top of age and gender (Manouchehrinia et al., 2020). sNfL is higher in patients with RRMS than in healthy controls and its increased levels indicate a risk for a clinical event in patients with the radiologically isolated syndrome (RIS) (Bittner et al., 2021). In another study, sNfL levels peaked 3–4 weeks after a clinical relapse and persisted elevated for 6–12 months. Besides, sNfL correlated with cortical atrophy and decreased spinal cord volume, even in absence of evidence of activity by MRI (Ferreira-Atuesta et al., 2021). Elevated sNfL levels have predictive value for future relapses, new T2 or gadolinium enhancing lesions and future spinal cord and brain atrophy (Bittner et al., 2021).

In a recent review of ongoing pharmacological clinical trials (31 protocols) in MS, only 5% of the interventional studies adopted fluid biomarkers as outcome measure in MS. Among these, determination of sNfL was widely used as a biomarker across all MS phenotypes with occasional determination in the CSF. Determination of glial fibrillary acid protein (GFAP), a soluble glial marker, in CSF, may assist in differentiating PPMS and RRMS in their early stages (Schilke et al., 2024). In another study, sNfL and GFAP but not ubiquitin c-terminal hydrolase L1 (UCHL1) and total Tau (tTau) serum concentrations were associated with CSF levels of the corresponding biomarker (Koerbel et al., 2024).

Although sNfL has gained reputation as a validated biomarker in the diagnosis and follow-up of patients with MS, this biomarker has also been linked with other neurological disorders that occur through lifespan including autism spectrum disorder (Öz et al., 2024), Huntington’s disease (Voysey et al., 2024); Alzheimer’s disease, traumatic brain injury, amyotrophic lateral sclerosis (ALS) and stroke (Park et al., 2024); fronto-temporal dementia (Katisko et al., 2020); Parkinson disease (Qi et al., 2024); cognitive decline (Desai et al., 2022), encephalitis related to SARS-CoV-2, HSV, and VZV (Wellmann et al., 2024), and insomnia (Ren et al., 2022). Plasma NfL predicted cerebellar atrophy and clinical progression in spinocerebellar ataxia (Coarelli et al., 2021). Determination of NfL in CSF of patients with HIV-associated dementia, ALS and Creutzfeldt-Jakob disease gave very high concentrations of the biomarker (Park et al., 2024). Since the increment of NfL reported in patients with encephalitis related to SARS-CoV2 was attributed to a possible dysregulation of the immune response, we may think that a similar mechanism could be involved upon EBV infection in patients who subsequently develop MS (Needham et al., 2022). In summary, sNfL is a sensitive biomarker of neurodegenerative, neuroinflammatory and neuropsychiatric disorders, but it is not specific to explain the mechanism of injury of the neuron cytoskeleton in each of the corresponding conditions.

### 2.4 Does CXCL13 play a role in the formation of the meningeal TLO?

How meningeal inflammation is precipitated during neurological disease and how to ameliorate that process for therapeutic purpose, is still a matter of investigation. Identification of clonally related B cells in the spinal fluid of MS patients suggests that there is an intrathecal differentiation of B cells which is independent of the periphery (VonBudingen et al., 2012). Lymphoid organogenesis and formation of mTLO could be facilitated by the expression of lymphotoxin-*α* in the external layer of the inflamed meninges, leading to a compartmental distribution of the immune response in MS. In the TLO, FDC play an important role in cell organization, B-cell recruitment, and production of CXCL13, which links the CXCR5 receptor expressed in a subtype of T-helper cells (Endres et al., 1999; Ansel et al., 2000; Cyster et al., 2000).

Meningeal stromal cells can produce CXCL13 in special circumstances leading to focal accumulation and organization of lymphoid cells in a specific site (Esen et al., 2014). Production of CXCL13 by infiltrating microglia and macrophages/DC myeloid cells was found in the model of neuroborreliosis in the macaque Rhesus (Ramesh et al., 2009). In addition, microglial cells with ability to produce CXCL13 were found in an animal model of neuroinflammation induced by the Sinbis virus (Esen et al., 2014). Intrathecal production of CXCL13 was found decreased in patients with non-inflammatory neurological conditions, high in patients with Lyme neuroborreliosis (>250 pg./mL) and moderate high in patients with MS who had focal dysfunction of the blood-CSF barrier (5–100 pg.//mL) (Pachner et al., 2024). ICXCL13 has been useful in the follow-up of patients with primary lymphoma of the CNS during diagnosis and after treatment (Pachner et al., 2024). Sensitive methods for determination of CXCL13 are available as the Bead-based assay (Luminex or SIMOA), which requires processing at specialized laboratories (Pachner et al., 2024). Besides, CXCL13 is an extremely stable biomarker that has been identified in samples after storage for prolonged time (Pachner et al., 2025).

The B-cell repertoire found in the meningeal TLO was different from those found in the SLO suggesting that the meningeal TLO represents sites of independent immune activity and that their GC activity is antigen driven (Lehmann-Horn et al., 2016). In an animal model of experimental allergic encephalitis (EAE) with a deficiency of CXCL13 the course of disease was benign despite inflammation of the white matter and gliosis during the acute and chronic phase (Bagaeva et al., 2006).

A current challenge in MS research is the identification of patients with higher degrees of meningeal inflammation and a high likelihood of formation of TLO. Higher levels of CXCL13, CXCL12, IFN-*γ*, TNF, sCD163, LIGHT and APRIL were found in CSF, 4 years after follow-up, in a cohort of RRMS naïve patients who had experienced evidence of disease activity (Magliozzi et al., 2020). In this study, a strong correlation between CXCL13 levels in CSF and development of new cortical lesions was found by MRI, thus, demonstrating that CSF profiles are useful to characterize patients at greater risk of cortical lesion accumulation (Magliozzi et al., 2020). An increased expression of inflammatory cytokines such as IFN-γ and TNF-*α* and chemokines/cytokines related to lymphoid neogenesis such as CXCL13, CXCL10, IL-6 and IL-10, was found during a determination of CSF profiles collected before death in MS cases with known TLO (Magliozzi et al., 2018).

### 2.5 What is the relevance of the CXCL13 index in multiple sclerosis?

The CXCL13 index (ICXCL13), was identified as a potential biomarker of intrathecal production of CXCL13 to predict future disease activity in MS patients 2 years after presenting with the clinically isolated syndrome (CIS) (Pike et al., 2023). In their study, patients with CIS or RIS underwent a CSF and blood specimen collection and ICXCL13 was determined. After a 5-year period of follow-up, patients with high ICXCL13 were more likely to convert to clinically definite MS (82.4%) compared to those with low ICXCL13 (10.0%). This predictive ability was consistent in CIS and RIS patients for at least 5 years. This data supports the concept that ICXCL13 has the potential to be used as a guide to immunomodulatory therapy in MS (Pike et al., 2023). This study also found that CIS patients responded well to therapy with moderate efficacy-DMT or no DMT when the ICXCL13 was low, opposite to patients who had a high ICXCL13 (Pachner et al., 2024). In another study, Alvarez et al. reported a significant drop in the ICXCL13 in patients who were treated with add-on rituximab (Alvarez et al., 2015). In MS patients, the CXCL13 outperformed both OCB and CSF NfL values in prediction ability. For ICXCL13, the positive predictive value (PPV) was 69% and the negative predictive value (NPV) was 89%, with higher sensitivity and specificity for predicting future disease activity (Disano et al., 2020). In analyzing only CIS patients, ICXCL13 also proved to be a better predictor of disease, with a sensitivity of 90%, a specificity of 56%, PPV of 53% and NPV of 91% (Disano et al., 2020). To our knowledge, ICXCL13 was not determined in specimens from any MS patients participating in clinical trials with therapeutic agents.

CXCL13 concentrations in CSF have been found in early active disease and in progressive MS. CXCL13 is not likely to enter the CSF from the bloodstream despite its small molecular weight, even in the presence of BBB alterations, thus, suggesting that elevated CXCL13 in CSF may be indicative of a neuroinflammatory process. Determination of serum concentrations of CXCL13 is not specific as a biomarker for intrathecal production since this chemokine may show high levels in patients with systemic autoimmune, inflammatory, infectious or neoplastic disease (Di Filippo et al., 2024; Pilz et al., 2020).

### 2.6 Would it be possible to visually identify the mTLO in patients with multiple sclerosis, in-vivo?

Leptomeningeal contrast enhancement (LMCE) has been identified as an eventual marker of meningeal inflammation in MS, which was confirmed in RRMS, SPMS and primary progressive MS (PPMS) using different magnetic field magnitude (3 T and 7 T) (Kee et al., 2022). LMCE has also been associated with gray matter atrophy, longer disease span and increment of neurological disability even though it does not represent the TLO per se (Kee et al., 2022). Bhargava et al. were able to identify LMCE lesions in the T1 weighted and FLAIR sequences using an animal model of EAE that entailed meningeal inflammation and TLO formation, and by means of a 11.7 Tesla scan. LMCE was seen on the surface of the cortex and hippocampal fissure in MRI, which matched areas of meningeal TLO that expressed FDC M1 biomarkers, PNAD (endothelial venules marker found in lymphoid tissue) and CXCL13. Similarly, changes in the subjacent cortex were characterized by microglial and astrocytic activation, demyelination and axonal damage (Bhargava et al., 2021). This study also reported reduction of LMCE and reduction of B cells percentage in the TLO after therapy with the BTK inhibitor (evobrutinib) (Bhargava et al., 2021).

In a case–control study of MS patients and healthy controls, Harrison et al. demonstrated that post-contrast 3D FLAIR images, acquired at a 7 Tesla MRI with gadolinium (27 min after injection), were the most sensitive sequence for LMCE, with no difference between the groups and being associated with age (Harrison et al., 2024). LMCE was also present in Alzheimer and mild cognitive impairment (Freeze et al., 2017), traumatic brain injury, and cerebral ischemia (Freeze et al., 2020; Wu et al., 2021; Ineichen et al., 2022), which are conditions not necessarily associated with neuroinflammation (Harrison et al., 2024).

### 2.7 Could disease modifying therapy play an inhibiting role in the formation of the TLO?

The neutralization of the TLO could play a paramount role in the therapy of MS by blocking the re-emergence of autoreactive clones with ability to trigger flare episodes or resistance to therapy (Pitzalis et al., 2014). In mouse models of EAE and opticospinal encephalomyelitis, administration of the anti-mouse CD20 clone 18B12 intraperitoneally led to depletion of B-cells in the periphery (blood and lymph nodes) but the TLO were able to develop in the CNS without modification in the course of progressive disease, respectively (Brand et al., 2021; Mitsdoerffer et al., 2021). The administration of intrathecal rituximab and intravenous ocrelizumab, in two different studies in MS patients, failed to prevent LMCE (Bhargava et al., 2019; Zivadinov et al., 2022). In two EAE studies, siponimod, a modulator of the sphingosine-1-phosphate receptors 1,5 that induces lymphopenia and is known to cross the BBB, was able to reduce the formation of the TLO (Brand et al., 2022; Gentile et al., 2016).

A study with a nucleoside drug, cladribine (2-chlorodeoxyadenosine), conducted 30 years ago, documented a reduction in concentration of OCB in CSF of patients receiving this drug opposite to placebo (Sipe et al., 1994). Recently, oral cladribine was found to primarily deplete non-class switched memory B cells and antibody-secreting cell precursors in RRMS leading to long term reduction (up to 96 weeks) in intrathecal antibody production, and reduction of the IgG index, the kappa free light chain (kFLC) index and the CXCL13 in the CSF (Ammoscato et al., 2024).

CSF concentrations of CXCL13 decreased after therapy with autologous hematopoietic stem cell transplant (AHSCT) in patients with RRMS at the first follow-up at 1 year than at baseline (Lundblad et al., 2023), and after therapy with rituximab, natalizumab and high-dose methylprednisolone in patients with CIS, RRMS, PPMS and SPMS (Serafini et al., 2004; Irani, 2016; Sellebjerg et al., 2009).

## 3 Conclusion

In MS, mTLO are lymphoid structures that originate and persist in the meninges and have the capacity to harbor and generate proinflammatory and autoreactive cells that perpetuate disease (Shipman et al., 2017). Since the presence of ICXCL13 indicates the existence of meningeal TLO, high efficacy therapy (HET) given at time of diagnosis of multiple sclerosis should prevent further formation of mTLO, thus, halting progression of disease and improving prognosis, especially in patients with RIS and CIS with high ICXCL13. The application of biomarkers, such as the ICXCL13 could significantly help to accomplish this endeavor although special awareness about the adverse effects of immunosuppression must be observed.
